# 

**Published:** 1866-10

**Authors:** 


### The Chicago Medical Journal.

**E. L. HOLMES, M.D., H. M. LYMAN, M.D., R. M. LACKEY, M.D. . . . Editors.**

All letters for the JOURNAL should be addressed to R. M. LACKEY, M.D., P. O. Box 2175, Chicago, Ill. *Office—169 South Dearborn Street.*

**TERMS—$2 PER ANNUM, IN ADVANCE.**

**$3 if not paid before 1st of July of each year. Single Copies, 25 cents.**

### PAMPHLETS RECEIVED.

On Excision of the Superior Maxilla: Report of a case by Wm. R. Whitehead, M. D., New York. 1866.

Asiatic Cholera.—Abstract of a report to the International Sanitary Conference at Constantinople. Reprinted from the *Boston Medical and Surgical Journal*, for Aug. 9, 1866.

Transactions of the Vermont Medical Society, for the year 1865.

L'Art Dentaire. A Monthly Review of the Science and Art of Dentistry. Chief Editor, A. Preterre, 29 Boulevard des Italiens, Paris. July, 1866.

---

It would be difficult for Ticknor & Fields to improve the *Atlantic Monthly*, but we notice that each issue of *Our Young Folks* is now adorned with a full page illustration, making it one of the most fascinating monthlies of the season. *Every Saturday* has also been enlarged, and improved by the introduction of a serial tale among its other well selected extracts from the most popular European periodicals.

